# Mycoplasma Pneumoniae with Atypical Stevens-Johnson Syndrome: A Diagnostic Challenge

**DOI:** 10.1155/2013/457161

**Published:** 2013-01-08

**Authors:** Ralph Yachoui, Sharon L. Kolasinski, David E. Feinstein

**Affiliations:** Division of Rheumatology, Cooper Medical School of Rowan University, Suite 262, E and R Building, The Haddon Avenue Office, Camden, NJ 08103, USA

## Abstract

The Stevens-Johnson syndrome (SJS) classically involves a targetoid skin rash and the association of the oral mucosa, genitals, and conjunctivae. Recently, there have been several documentations of an incomplete presentation of this syndrome, without the typical rash, usually associated with the mycoplasma pneumoniae infection. Our case illustrates that this important clinical diagnosis should not be missed due to its atypical presentation.

## 1. Introduction

Mycoplasma pneumoniae (MP) is a common cause of community-acquired pneumonia in young adults [1]. Extrapulmonary manifestations occur in <10% of cases and include hemolytic anemia, hepatitis, arthritis, meningitis, and Stevens-Johnson syndrome (SJS) [2].

In the recent literature, there have been few reports of isolated mucositis associated with MP infection, labelled as atypical SJS, mostly in children with ages ranging from 8 to 21 years [1, 3].

Here, we report a case of MP-associated mucositis with the absence of skin lesions in a previously healthy adult male.

## 2. Case

A 29-year-old African American male, with no past medical history, was admitted to our institution with oral and genital ulcers for 3 days. One week prior to admission, he developed a flulike illness with congestion and cough. His review of systems was significant for odynophagia. He admitted cocaine use.

**Figure 1:**
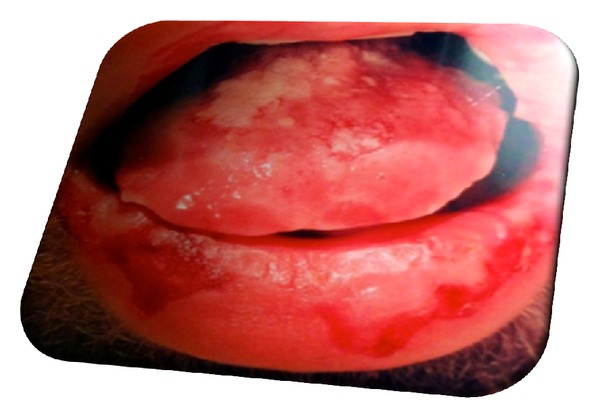
Swollen crusted lips.

**Figure 2:**
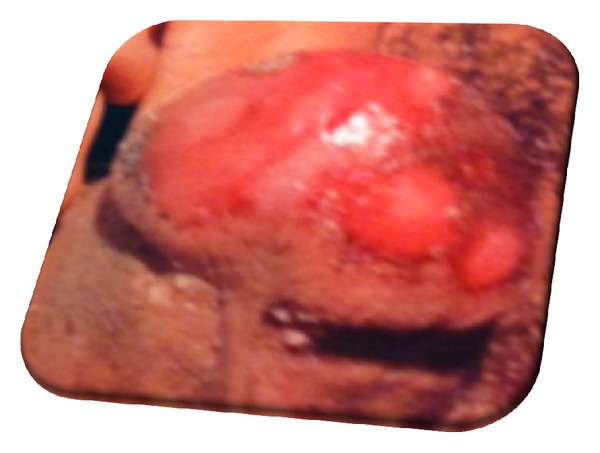
Erosive lesions on the glans penis.

Physical examination showed markedly injected conjunctivae bilaterally, with swollen, crusted lips (Figure 1). The mouth and pharynx had diffuse ulcerations and scattered hemorrhagic areas. The genitalia had erosive lesions on the glans penis (Figure 2). No skin rashes. 

Laboratory investigations revealed negative throat HSV PCR, throat gonococcus/chlamydia PCR, syphilis, and HIV test. The pathergy test was also negative. Mycoplasma pneumoniae IgM titer was positive on enzyme immunoassay. The diagnosis of atypical Stevens-Johnson syndrome due to mycoplasma pneumonia infection was made.

Patient was started on solumedrol at a dose of 40 mg intravenous twice daily. His symptoms improved quickly and he was discharged home two days later.

## 3. Discussion

Mycoplasmas are ubiquitous and are the smallest, free-living microorganisms. After an incubation period of 1 to 4 weeks, infection typically presents with cough, pharyngitis, and rhinorrhea. Only 10% of patients develop pneumonia [4]. Extrapulmonary manifestations of MP infection are unusual and include SJS, arthritis, hemolytic anemia, and encephalitis [2]. Serology is the mainstay of laboratory diagnosis. When available, the polymerase chain reaction (PCR) is a rapid and helpful test, especially when combined with serology [5].

Stevens-Johnson syndrome (SJS) is thought to fall within a spectrum of diseases that affect the skin and mucous membranes, including erythema multiforme minor, erythema multiforme major (or SJS), and toxic epidermal necrolysis [6]. Mucosal ulcerations typically occur at 2 or more sites such as oral, ocular, or genitourinary. The skin lesions are often quite targetoid. Painful erosions may extend into the esophagus and cause difficulty with swallowing [6]. Medications are the leading trigger of SJS in adults, whereas infections are the most commonly identified cause in children [2]. Multiple retrospective reports showed that MP infection may be the most common infectious agent associated with SJS [2, 7].

MP-associated mucositis with the absence of skin lesions is extremely rare and has been reported in children. Many authors now agree that this is a separate entity from SJS, labelled as atypical SJS [3, 8]. As in SJS, the pathogenesis is unclear, although it has long been suggested that immunological mechanisms may play an important role [1]. Making the diagnosis of atypical SJS is clinically challenging, given that a number of other diseases including autoimmune diseases and infections can manifest with mucosal changes. Our case is unique as it occurs in an adult patient.

The treatment of SJS is supportive with nutrition, fluids, pain management, and withdrawal of the offending agent when known. Antibiotics may have a role in treating MP infection. The role of steroids in SJS remains controversial [3]. Our patient improved quickly after intravenous solumedrol, supporting the underlying immune mediated nature of the disease.
